# QTc Dynamics Following Cardioversion for Persistent Atrial Fibrillation

**DOI:** 10.3389/fcvm.2022.881446

**Published:** 2022-06-03

**Authors:** Arwa Younis, Nofrat Nehoray, Michael Glikson, Christopher Bodurian, Eyal Nof, Nicola Luigi Bragazzi, Michael Berger, Wojciech Zareba, Ilan Goldenberg, Roy Beinart

**Affiliations:** ^1^Cardiac Electrophysiology and Pacing Section, Department of Cardiovascular Medicine, Cleveland Clinic, Cleveland, OH, United States; ^2^Chaim Sheba Medical Center Affiliated to Sackler Medical School, Tel Aviv University, Ramat Gan, Israel; ^3^Heart Center, Shaare Zedek Medical Center, Jerusalem, Israel; ^4^Clinical Cardiovascular Research Center, University of Rochester, Rochester, NY, United States; ^5^Laboratory for Industrial and Applied Mathematics, Center for Disease Modeling, York University, Toronto, ON, Canada

**Keywords:** persistent atrial fibrillation, cardioversion, safety, QT interval, QTc prolongation

## Abstract

**Background:**

Cardioversion (CV) for atrial fibrillation (AF) is common. We aimed to assess changes in QTc over time following electrical CV (ECV) for persistent AF, and to compare the benefit of using continuous Holter monitoring vs. conventional follow-up by ECG.

**Methods:**

Prospective observational cohort study. We comprised 90 patients admitted to our center for elective ECV due to persistent AF who were prospectively enrolled from July 2017 to August 2018. All patients underwent 7-days Holter started prior to ECV. Baseline QTc was defined as median QTc during 1 h post ECV. The primary endpoint was QTc prolongation defined as QTc ≥500 ms, or ≥10% increase (if baseline QTc was >480 ms). Conventional monitoring was defined as 2-h ECG post ECV.

**Results:**

Mean age was 67 ± 11 years and 61% were male. Median baseline QTc was 452 ms (IQ range: 431–479 ms) as compared with a maximal median QTc of 474 ms (IQ range: 433–527 ms; *p* <0.001 for the change in QTc from baseline). Peak median QTc occurred 44 h post ECV. The primary endpoint was met in 3 patients (3%) using conventional monitoring, compared with 39 new patients (43%) using Holter (*p* <0.001 for comparison). The Holter monitoring was superior to conventional monitoring in detecting clinically significant QTc prolongation (OR = 13; *p* <0.001).

**Conclusions:**

ECV of patients with persistent AF was associated with increased transient risk of QTc prolongation in nearly half of the patients. Peak median QTc occurs during end of second day following ECV and prolonged ECG monitoring provides superior detection of significant QTc prolongation compared with conventional monitoring.

## Introduction

Atrial fibrillation (AF) is the most common sustained arrhythmia worldwide and represents a major challenge in patients' management (1).

Rhythm control is the preferred strategy in patients with symptomatic AF. Electrical cardio-version (ECV) and pharmacological cardio-version (PCV) are widely used in order to restore sinus rhythm (SR). ECV is more effective than PCV, particularly in persistent AF (2), and while proven to be a safe, in some cases it could lead to serious adverse events, including QT prolongation and Torsade de pointes (3, 4).

It has been suggested that persistent AF induces ventricular repolarization remodeling leading to transient QT prolongation following ECV (5). This might increase the risk for Torsade de pointes, especially when other QT prolonging conditions exists (4). Furthermore, the European Heart Society and the American Heart Association guidelines recommend the initiation of antiarrhythmic drug therapy 1–3 days before ECV to promote sustainable cardioversion (6, 7). In fact, many of these drugs have a potential of further QTc prolongation, increasing the risk even more (8). Currently, there are no guidelines specifying the time needed to monitor patients following ECV. The common practice is to watch the patients for 1–2 h following ECV, and thereafter to discharge them.

We hypothesized that patients with persistent AF on antiarrhythmic treatment might develop significant QTc prolongation following ECV during long-term monitoring. Therefore, in this prospective clinical study we aimed to (1) assess changes in QTc following ECV and to identify the time to maximal QTc prolongation, (2) to compare the current standard of care to 7-days Holter monitoring, and (3) to identify clinical predictors for clinically significant QTc prolongation.

## Methods

### Study Patients

**Figure 1:**
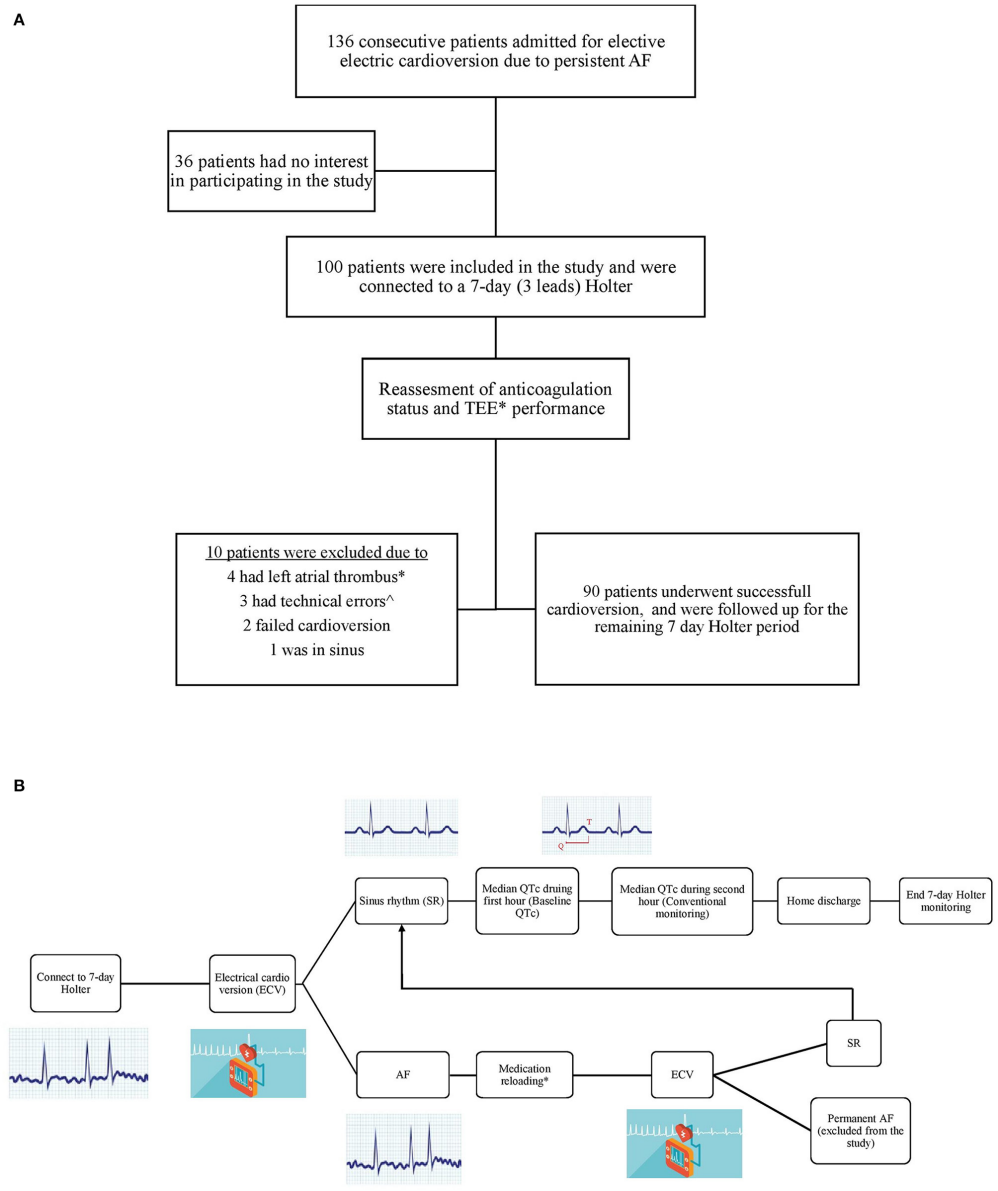
**(A)** Study flow chart. **(B)** Patients management chart flow. *Trans esophageal echocardiography (TEE) was performed in two third of the patients due to their high risk profile. ∧Two patients had very bad recordings quality, and in one patient lead three was disconnected prior to the CV and remained disconnected thereafter.

From 09/07/2017 to 27/08/2018, we prospectively enrolled consecutive patients with persistent AF who were admitted to the cardiology ward at the Chaim Sheba Medical Center (Tel Hashomer, Israel) for electiveECV. Our study inclusion criteria were: (1) age ≥18 years old, (2) symptomatic persistent AF/AFL (atrial fibrillation or atrial flutter), (3) baseline QTc >300 ms, (4) no contraindication for ECV, and (5) no contraindication for anticoagulation. Study exclusion criteria included: (1) recent initiation of medication that is well-known to prolong QTc, (2) recent increase in dose of potentially prolonging medication (other than the medication desired for the ECV), (3) pregnancy, (4) patients with congenital long QT syndrome, (5) baseline QTc >500 ms, and (6) history of Torsade de Points. Out of the 136 consecutive patients admitted to the award who met the inclusion criteria, 100 patients agreed to participate in the study. All enrolled patients were connected to a 7-day 3-lead Holter prior to ECV (in order to monitor QTc during AF) (Figure 1A).

All patients underwent transesophageal echocardiography (TEE) if effective anticoagulation status was not confirmed or upon physician discretion. In addition, all underwent transthoracic echocardiography (TTE) within 3 months prior to the study enrollment, or on the day of ECV. Of note, after connecting patients to Holter, ten patients (10%) were withdrawn from the study due to left atrial appendage thrombus or severe swirling in TEE (4 patients), Holter malfunction (3), failure to cardiovert to sinus rhythm (2) and a patient who spontaneously converted to sinus rhythm following TEE. All remaining 90 patients were successfully electrically cardioverter to sinus rhythm and were monitored according to the study protocol using the Holter for 7 days.

The protocol was approved by the institutional review board of the Chaim Sheba medical center (2711-15-SMC) affiliated to the University of Tel Aviv. All patients provided written informed consent. The primary hypothesis was that signifcant QTc prolongation can occur following ECV during 7 days of prolonged ECG monitoring, and that the use of Holter would be superior to the conventional protocol in detecting potentially clinically significant QTc prolongation.

### Cardioversion

All patients were on antiarrhythmic drugs at baseline (Amiodarone 200 mg once daily, Sotalol 80–120 mg twice daily, Flecainide 100 mg twice daily, or Propafenone 150–225 mg three times daily). After light sedation with Midazolam (up to a maximum of 5 mg, based on the patient weight), all patients underwent ECV with 200 Joules using a biphasic defibrillator patches. If sinus rhythm was not achieved and upon discretion of the treating physician, antiarrhythmic drugs were given for 4–12 h (Amiodarone 900–1,200 mg, Flecainide 400 mg, Propafenone 450 mg). After several hours (12 h for Amiodarone, 4 h for Flecainide and Propafenone) an ECG was performed. If the patient was still in AF, ECV shock was re-delivered using the same sedation and shock protocol as described above. After achieving SR, patients were monitored for 2 h (conventional monitoring) and thereafter discharged home with Holter monitoring (Figure 1B). Patients were seen in office for clinical follow-up at 1–3 months following ECV.

### QTc Measurement

The methods of computerized QT analysis and their reproducibility have been described in detail previously (9, 10). For this study, a 3 lead Life card CF (OSI systems) Holter was used and data were analyzed using the Spacelab Pathfinder SL software with measurements in lead number II. The Bazett formula was used to obtain heart rate-corrected values of parameters of QTc interval. Median QTc was given hourly. All measurements were adjudicated by electrophysiology fellow (AY) and by internal Emergency Medicine resident (NN). If a change between two consecutive measurements, calculated as $\frac{QTc\;second-QTc\;first}{QTc\;first}\times 100$, was >10%, a manual validation/correction was performed. Furthermore, manual adjustment was made for patients with pacemaker during pacing periods. In order to minimize the errors that may occur during short tachy-arrhythmic events, or secondary to diurnal variability, the median of every 4 h values was used. Accordingly, each patient had 6 measurements per day. Examples are listed in the Supplementary Figure A. In order to validate the results further, 10 random Holter strips were printed and evaluated blindly by a senior electrophysiologist (RB), and a fellow (AY), and the results were compared with the computerized results. The matching rate was 99.7%. Examples are listed in the Supplementary Figures B,C.

### Definitions and Endpoints

Baseline QTc was defined as the median QTc during the first hour post ECV. Conventional monitoring was defined as the Holter tracing at the second hour following ECV. Holter monitoring was defined as tracings starting from the beginning of the third hour following ECV throughout the end of the monitoring period. The primary endpoint was clinically significant QTc prolongation defined as; (1) new prolongation of QTc ≥500 ms (if baseline QTc was <480 ms) or (2) prolongation of QTc ≥10% if baseline QTc was >480 ms (11).

### Statistical Analysis

Continuous variables are expressed as mean ± SD. Categorical data are summarized as frequencies and percentages. All variables were tested for normality using four tests; the Shapiro-Wilk (Shapiro & Wilk, 1965), the Kolmogorov-Smirnov test, Cramer von Mises test, and the Anderson-Darling test. All QTc values (during AF, after 2 h, and max) were all non-normal distributed. Therefore, we report median QTc instead of mean QTc. The test that was used to compare the medians was Wilcoxon rank Sum test. Median QTc baseline (first hour post ECV), median QTc during the second hour (conventional monitoring), and the median QTc every 4 consecutive hours (Holter monitoring) were displayed in a graph with 25%−75% confidence interval, while the Y axis is the QTc, and X axis is time from ECV.

We included 18 potential clinical, electrocardiographic, echocardiographic and laboratory binary risk factors for QTc prolongation (Supplementary Table A). Numeric variables were made binary by the use of cut points with the goal of finding simple, easily implemented predictors to be derived from them. Thresholds for categorization of numeric variables were based on the mean value. Univariate relationships between candidate covariates and a further event were assessed by *t* tests (2 for binary responses). The covariates with values of *P* <0.10 were further evaluated by carrying out a best-subset regression analysis, examining the models created from all possible combinations of predictor variables, and using a penalty of 3.84 on the likelihood ratio 2 value for any additional factor included (corresponds to a *P* of 5% for a 1-df 2 test).

Detection rates were calculated as a fraction of all patients who had received 7-days Holter monitoring. The cumulative probability of AF was displayed according to the Kaplan-Meier method. The differences between detection rates for different monitoring intervals within each patient were tested using McNemar's test as appropriate. All statistical tests were two-sided, a *p*-value of <0.05 was considered statistically significant. Analyses were carried out with SAS software (version 9.4, SAS institute, Cary, North Carolina).

## Results

The baseline clinical characteristics of study patients are shown in Table 1. The mean age of the study cohort was 67 ± 11 years, 55 patients (61%) were male. The mean CHA_2_DS_2_VASc was 3.5 ± 1.5. Baseline mean heart rate in AF was 80 ± 20 bpm and median was 76 (IQR 64–92). In 7 patients (8%), ECV was performed due to persistent atrial flutter. Most patients had hypertension 60 (67%) and were likely to receive novel oral anti-coagulants 66 (73%). Amiodarone was the most common antiarrhythmic medication used prior to ECV (60 patients, 67%). Despite the fact that the vast majority of patients were on adequate anti-coagulation status, TEE was performed in 63 (70%) patients prior to CV, due to increased stroke risk in this population.

**Table 1:** Baseline characteristics.

	**Study cohort (No. = 90)**	**Study cohort**		***P* value**
		**Significant QTc prolongation**		
		**No (No. = 48)**	**Yes (No.42)**	
Male, %	55 (61)	26 (60)	27 (61)	0.83
Age, mean (SD)	67 ± 11	67 ± 12	67 ± 11	0.91
BMI, mean (SD), kg/m2	30 ± 5	29 ± 4	30 ± 5	0.44
Atrial flutter	7 (8)	5 (11)	2 (4)	0.25
Heart rate in AF, mean (SD), bpm	80 ± 20	78 ± 21	82 ± 20	0.56
Ischemic heart disease, %	19 (21)	8 (16)	12 (27)	0.30
CHF, %	30 (36)	12 (29)	18 (43)	0.25
TEE, %	63 (70)	31 (63)	32 (76)	0.67
EF, mean (SD), %	52 ± 12	54 ± 12	49 ± 14	0.11
LA size, mean (SD), cm	4.5 ± 0.4	4.5 ± 0.5	4.4 ± 1	0.64
Mitral regurgitation, %	56 (62)	23 (48)	33 (79)	0.049
Mitral stenosis, %	14 (15)	7 (15)	7 (17)	0.86
Aortic stenosis, %	12 (13)	6 (14)	6 (15)	0.87
SPAP, mean (SD), mmHg	34 ± 8	34 ± 8	35 ± 7	0.75
Diabetes, %	25 (28)	9 (19)	16 (36)	0.093
Hypertension, %	60 (67)	29 (67)	30 (68)	0.83
Renal disease, %	21 (23)	10 (24)	11 (25)	0.85
CHA_2_DS_2_VASc, mean (SD)	3.5 ± 1.5	3.3 ± 1.6	3.7 ± 1.7	0.32
ICD/CRTD, %	2 (2)	None	2 (4)	0.49
Pacemaker	11 (12)	5 (11)	6 (15)	0.74
ACE Inhibitor or ARB, %	47 (52)	22 (46)	25 (60)	0.64
Aldosterone, %	10 (11)	4 (9)	6 (15)	0.74
Beta-blocker, %	67 (74)	30 (63)	37 (88)	0.04
Calcium channel blocker, %	25 (28)	12 (26)	13 (29)	0.81
Digitalis, %	6 (7)	2 (4)	4 (9)	0.67
Amiodarone, %	60 (67)	30 (63)	30 (69)	0.83
Flecainide, %	20 (22)	10 (21)	10 (23)	0.79
Propafenone, %	5 (6)	4 (9)	1 (2)	0.36
Statins, %	39 (43)	21 (46)	18 (42)	0.56
NOAC, %	66 (73)	33 (69)	33 (76)	0.47
Warfarin, %	24 (27)	14 (30)	10 (23)	0.41
Creatinine, mean (SD), mg/dl	1 ± 0.3	1 ±0.3	0.9 ± 0.3	0.32
K, mean (SD), mg/dl	4.3 ± 0.4	4.3 ± 0.4	4.3 ± 0.4	0.73
Na, mean (SD), mg/dl	140 ± 4	140 ± 3	140 ± 2	0.49
Mg, mean (SD), mg/dl	2 ± 0.4	2± 0.2	2 ± 0.4	0.94

*AF, atrial fibrillation; ACE, angiotensin converting enzyme; ARB, angiotensin receptor blocker; BMI, body mass index; CHF, congestive heart failure; COPD, chronic obstructive pulmonary disease; CRTD, cardiac resynchronization therapy with a defibrillator; ICD, implantable cardioverter defibrillator; EF, ejection fraction; LA, left atrial dimension; NOAC, novel oral anti-coagulant; SPAP, systolic pulmonic arterial pressure; TEE, trans-esophageal echocardiography*.

A total of 36 patients (40%) were treated with oral antiarrhythmic medication loading prior to the scheduled ECV (72% with amiodarone, and 28% with Class IC). Median max QTc was 537 (IQR 472–590) in the loading group and did not differ significantly from the no-loading group [522 (IQR 475–555); *p* = 0.32; Table 2].

**Table 2:** QTc values and atrial fibrillation recurrence rates based on the loading and re-loading of antiarrhythmic drugs.

	**All patients (*****N*** **= 90)**			**All patients (*****N*** **= 90)**		
	**Prior loading of AAD (*N* = 36)**	**No loading of AAD (*N* = 54)**	***p*-value**	**Reloading (*N* = 11)**	**No reloading (*N* = 79)**	***p*-value**
Amiodarone	26 (72)	NA		7 (64)	NA	
Flecanide/propafenone	10 (28)	NA		4 (36)	NA	
Max QTc prior to ECV	465 (446–500)	466 (440–494)	0.36	454 (424–483)	466 (445–500)	0.49
2 h post ECV	451 (433–471)	465 (432–500)	0.76	463 (422–494)	458 (433–494)	0.38
Max QTc	537 (472–590)	522 (475–555)	0.32	489 (451–523)	537 (481–580)	0.056
Patients with prologed QTc	22 (61)	24 (44)	0.13	2 (18)	43 (54)	0.066
Patients with AF recurrence	8 (22)	12 (22)	0.97	7 (64)	12 (15)	<0.001

*AAD, antiarrhythmic medications; ECV, electrical cardioversion; AF, atrial fibrillation/flutter*.

Eleven patients did not convert to SR with the first electrical shock. Re-loading of the same anti-arrhythmic medication, as described in the methods section, was performed in them (8 patients with Amiodarone and 3 with Flecainide). Among them, 8 patients (5 Amiodarone, 3 Flecainide) converted to SR with the second ECV. The remaining 3 patients needed a third ECV with 360 Joules in order to convert to SR.

### QTc Dynamics During Long-Term Holter Recording

**Figure 2:**
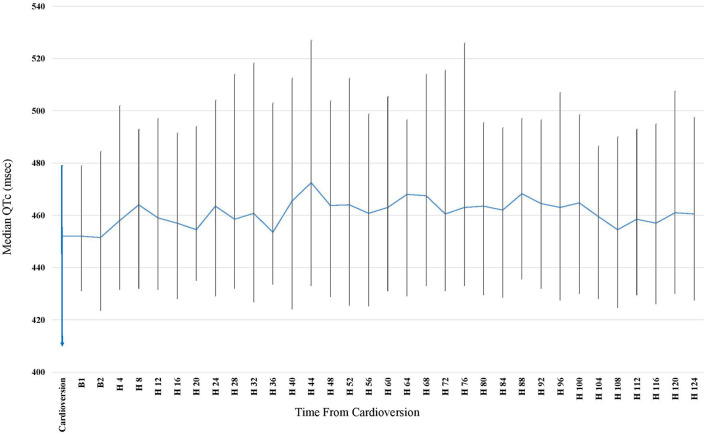
Median QTc obtained by Continuous 7 days Holter following Elective Cardioversion (CV) for Persistent Atrial Fibrillation. Time Zero Represents the CV Time. At time 132 h post CV, 33 (37%) of the patients were no longer connected to the 7-days Holter (due to longer detection prior to CV). The rate of patients connected to the Holter became lower every further hour beyond the 132 h post CV, making it incorrect to calculate for means and draw conclusions from it. Therefore, the graph applies only until 128 h post CV.

Patients underwent long term Holter monitoring for an average of 6.2 ± 1.2 days. Median baseline QTc (defined as the median QTc during first hour after ECV) was 452 (IQR 413–477) ms and was significantly lower than the median QTc during the continuous Holter monitoring [467 (IQR 432–497) ms, *p* = 0.01 for comparison]. The maximal median QTc occurred 44 h post CV and was 474 (IQR 439–511) ms (*p* <0.001 for comparison with baseline QTc). Following this peak, the QTc returned to the baseline QTc 132 h post ECV, with a median QTc of 458 (IQR 412–501) ms (*p* = 0.58 for comparison with baseline QTc) (Figure 2). The baseline clinical characteristics were similar between those who had significant prolongation within the 44 h post ECV and those who developed it after. A trend toward more Amiodarone use was seen in those who developed a later QTc prolongation (87% vs. 63%, *p* = 0.07). In 32 patients (35%), the median QTc reached new values ≥500 ms, and in 14 patients (16%) it even reached QTc ≥550 ms (baseline QTc was <480 ms).

### Independent Predictors of QTc Prolongation

In best subset forward regression analysis only prior beta blocker usage (HR 3.5, 95% CI 1.04–12.3, *p* = 0.042) was found to be independent predictor for potentially clinically significant QTc prolongation. Baseline QTc ≥ 450 was significant enough to enter the final model (*p* <0.10), however, in the final multiple regression model the *p*-value was not statistically significant (HR 2.2, 95% CI 0.89–5.2, *p* = 0.09) (Table 3).

**Table 3:** Predictors for clinically significant QTc prolongation 7-day Holter vs. 2-h conventional monitoring.

**End point**	**Hazard ratio**	**95% CI**	***p*-value**
**Clinically significant QTc prolongation (39 events)**			
Beta blocker	3.5	1.04-−12.3	0.042
Median QTc ≥ 450 during first hour post ECV	2.2	0.92-−5.2	0.093

During the 2 h following ECV (conventional monitoring), 3 (4%) patients met the primary endpoint. Among them, two patients had median QTc ≥ 500 ms (564 and 500 ms), and one had an increase of 11%. With the use of the 7-days Holter, significant QTc prolongation was detected in 39 patients (45%), significantly higher than the rate observed using the conventional monitoring period (*p* <0.001). Notably, among the 87 patients without clinically significant QTc prolongation detected using conventional 2-h monitoring, sixteen patients (18%) developed new QTc prolongation during the first day, 8 patients (11%) during the second day, and the rest thereafter. Importantly, as shown in Table 4, more than half of the QTc prolongation were detected within the first 2 days. Notably, during the second day, 5 patients had QTc > 550 ms (baseline QTc was <480 ms in all of them).

**Table 4:** Rates of clinically significant QTc during conventional monitoring and during 7-day Holter.

	**New Clinically Significant QTc Prolongation**	**Percentage of new events**	**No. of remaining patients without QTc prolongation**	**McNemar's *p* for comparison with conventional monitoring**
Conventional	3	3%	87	
First day	16	18%	71	0.0003
Second day	8	11%	63	0.025
Third day	7	11%	56	0.045
Fourth day	3	5%	53	NS
Fifth day	4	8%	49	NS
Sixth day	1	2%	48	NS
Seventh day	0	0%	48	NS
7 days Holter	39	43%	51	<0.001

**Figure 3:**
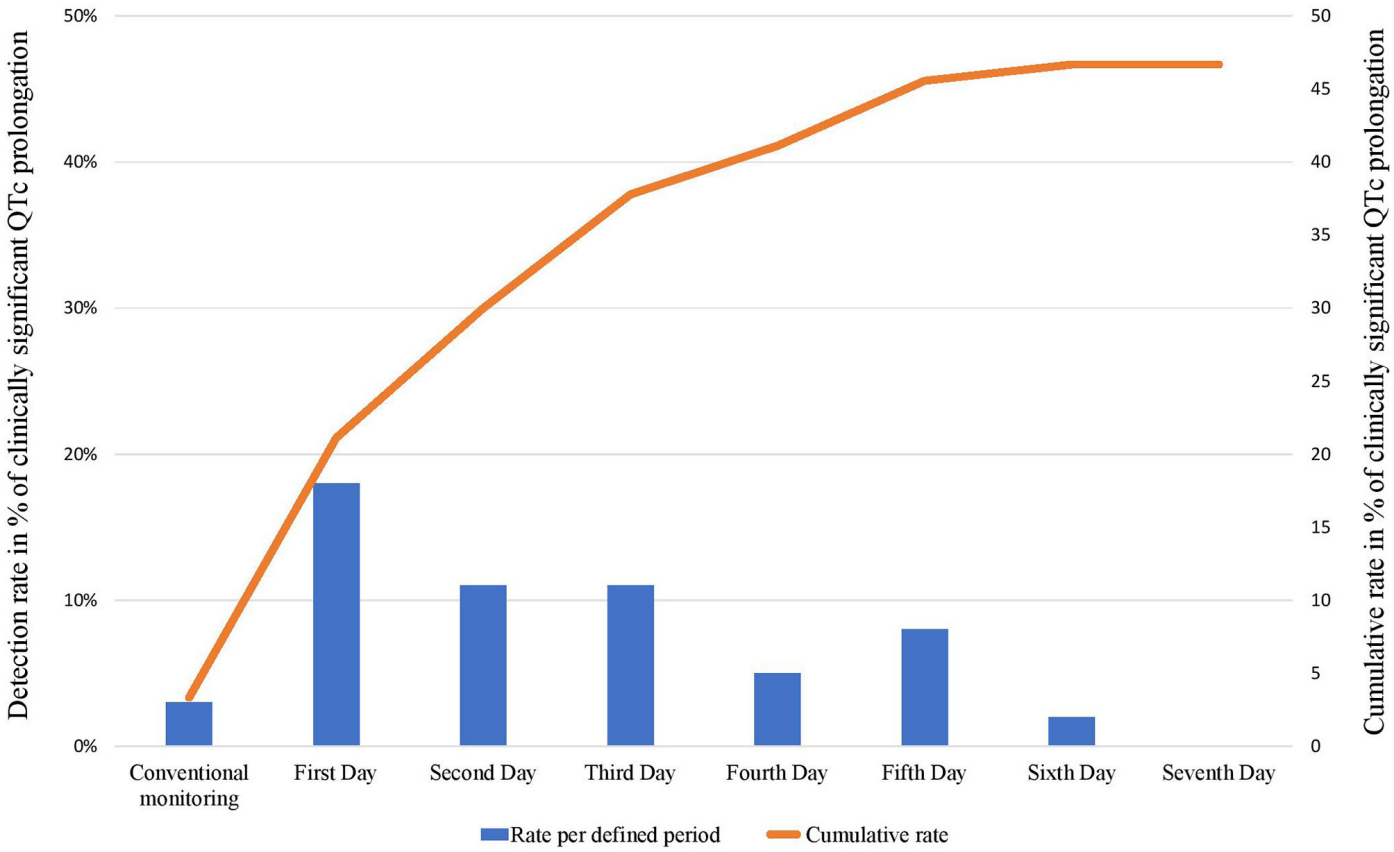
Daily/conventional (columns) and cumulative (line) detection rates of clinically significant QTc prolongation following cardioversion during 7 days of Holter monitoring.

Detection rates for every single day, and cumulative detection rates over the monitoring period are depicted in Figure 3. There was a strong recognizable pattern of detection rates favoring the first 48 h of the monitoring period.

Using McNemar test for comparison, the Holter monitoring was superior to conventional monitoring in detecting the PE with an OR of 13; 95% CI 5–65; *p* <0.001.

### Clinical Events and Death

During the study period, one patient died on the 5th day post ECV. The cause of the death was congestive heart failure exacerbation and pulmonary edema. This patient did not have clinically significant QTc prolongation. None of our patients had Torsade de points or sustained ventricular arrhythmia. No other significant clinical events were reported.

## Discussion

To the best of our knowledge this is the first observational prospective study that monitored closely the QTc interval following ECV in patients with persistent AF using 7-days Holter monitoring. The main finding of this study include (1) significant QTc prolongation that was detected in 47% of patients following ECV, (2) the maximal median QTc prolongation occurred during the second day (44 h following ECV), (3) there was a substantial increase in detection rates of clinically significant QTc prolongation using prolonged Holter monitoring compared with standard monitoring, and (4) chronic beta blockers and QTc ≥ 450 ms were the only predictors associated with significant QTc prolongation.

### QTc Prolongation Following ECV

In our study, during prolonged monitoring using 7-days Holter, we were able to demonstrate a significant number of patients with new significant QTc prolongation following ECV. Our observations are in line with previous case series and small studies (12, 13). Houltz and colleagues had previously reported QTc prolongation up to a mean of 672 ± 26 ms in patients that, in fact, developed Torsade de pointes (14). In another study Choy and colleagues reported that QTc was prolonged from 405 ± 55 to 470 ± 67 ms following ECV (3). These reports, however, included a relatively small number of patients (<10 patients in average) or included patients who were treated with antiarrhythmic drugs, that are known to prolong the QTc significantly, such as Sotalol or Dofetilide. Notably, in our study, patients with these antiarrhythmic medications (Sotalol or Dofetilide) were excluded, and all patients with other antiarrhythmic medication were already on these drugs more than 1 week before enrollment in the study. The exact mechanisms of QTc prolongation following ECV remain unclear. Possible causes include: (1) bradycardia—several studies demonstrated that lower heart rates following ECV may prolong the QTc interval (11), however, in our study the median HR was similar between those with significant QTc prolongation when compared to those without (Supplementary Table B), however this might be influenced by the AF recurrence in some patients, (2) increased drug toxicity—changes in the neuro-hormonal status, such as change in sympathetic nervous activity and ANP were reported following ECV (15), and can potentially modulate the sensitivity to antiarrhythmic drugs (3), abrupt slowing of heart rates, following termination of fast ventricular rates, that may lead to QTc prolongation (16), (4) ventricular repolarization remodeling during AF—QTc during SR following ECV was reported to be significantly and transiently prolonged [although of limited in magnitude, the prolongation was substantial (approximately 15%) in some individuals (5)], and (5) ion channels modification—in several previous studies (17) an alterations in ion channels and several gene expressions in the atria and ventricle of patients with persistent AF were reported leading to change in QTc interval; In particular, the inward rectifier I(K1) channel, affecting the inward rectifier potassium current which regulates the terminal portion of phase 3 of the repolarization (18). Midazolam use and regardless the need to additional use/reloading of antiarrhythmic medication peri-cardioversion.

### Holter Monitoring vs. Conventional Monitoring

Although the highest detection rate was observed during the first 24 h following ECV, a significant number of patients with QTc prolongation were detected thereafter. Furthermore, the maximum median QTc prolongation occurred during the second day (hour 44 post ECV), which attenuated thereafter, returning to baseline QTc. Similar findings were reported in a study that tested the QT/RR relationship following ablation of the atrioventricular junction in patients with AF. It demonstrated that the highest change in QTc was documented on the second day [(516 ± 51 ms on second day vs. 468 ± 26 ms baseline, *p* = 0.02) and (497 ± 37 ms on second day vs. 458 ± 25 ms baseline, *p* = 0.02)], afterwards the QTc normalizes with no statistical difference observed from days 3 to 7 at all heart rates (14). In light of our findings there might be a need for further monitoring beyond several hours post ECV in some individuals. In the past, and in accordance with this concept, the ACC/AHA/ESC 2006 Guidelines for the Management of Patients with AF recommended in hospital QT interval monitoring for 24–48 h following ECV in patients receiving drugs that prolong the QT interval. However, in the most recent AHA/ACC and ESC guidelines, the above-mentioned recommendations were omitted, and the monitoring time following ECV in patient on antiarrhythmic drugs became undefined. Hence, we believe that Holter monitoring or repeated ECGs are essential during the 48 h post ECV in some patients with persistent AF on antiarrhythmic drugs to identify patients with prolonged QTc.

### Predictors Associated With Significant QTc Prolongation

We found that chronic Beta blocker usage was associated with clinically significant QTc prolongation. Surprisingly, no other independent clinical or demographic characteristic was found to be associated with QTc prolongation. Previous studies have demonstrated controversy regarding the effect of beta blocker on the QT interval. Studies in patients with long QT demonstrated that at faster heart rates, beta-blockers shortened the maximum QT interval and resulted in shorter QTc, whereas at slower heart rates beta-blockers lengthened the maximum QT interval and resulted in longer QTc (19). We believe that these results are consistent with our findings as the prolongations of the QT interval were most likely to occur during relative bradycardia post ECV. Furthermore, in patients with AF, Beta blocker medication may be a marker of a more resistant or more progressive AF disease with rapid ventricular response, resulting in the need for treatment with Beta blockers for rate management. In our study, we failed to show association between previously reported risk factors [female gender, age, potassium level, and magnesium level (20)] and the subsequent risk for QTc prolongation. We believe this is mainly due to the small number of our cohort.

Our study has several limitations. The major limitation is that the majority of the patients were already on antiarrhythmic medication which may affect the QTc duration. Yet these medications were baseline medication and were not initiated during the study. Another important limitation is the lack of control group which plays an important role in such a study. The detection of a higher percentage of patients with QTc prolongation over time might be caused by physiological QT variability, together with long observational period, yet we aimed to assess the benefit of further monitoring per patient, using the conventional monitoring period as our control measurement. Importantly, in our study, some patients had in addition to their AF an abnormal conduction system (bundle branch blocks or complete block and were therefore paced), which may have prolonged the QTc interval. Nevertheless, the use of individualized comparisons (patient level analysis—holter vs. baseline) allows to adjust for these cofounders, which are found also at baseline and not only during the holter recording. Some of the patients had AF recurrence (whether transient or persistent) which may affect the accuracy of the QTc calculation made by the software (using the Bazzet formula which may overestimate the QTc during tachycardia), however, we educated all the results with delta of more than 10%, and we used a 4 h median to correct for short episodes of tachyarrhythmia's or bradyarrhythmias. Data on EHRA score or AF duration was not captured, however all patients were symptomatic patients with persistent AF.

Unfortunately, the vast majority of our patients did not attend the 1–3 months office visit. Follow-up was performed remotely using the phone and addressed questions regarding clinical events (hospitalization, health care utilization for arrhythmia, and/or death). ECG was performed only in 13 patients. Information on QTc on the long term remained unknown. However none of our patient has reported on health care utilization for arrhythmia or other arrhythmia related hospitalizations.

## Conclusion

ECV of patients with persistent AF was associated with increased transient risk of QTc prolongation in nearly half of the patients. Peak median QTc occurred during end of second day following ECV and prolonged ECG monitoring (by Holter or repeated ECGs) provided superior detection of significant QTc prolongation compared with conventional monitoring. In our study, this transient QTc prolongation did not result in clinical significant events.

## Data Availability Statement

The raw data supporting the conclusions of this article will be made available by the authors, without undue reservation.

## Ethics Statement

The studies involving human participants were reviewed and approved by University of Tel Aviv. The patients/participants provided their written informed consent to participate in this study.

## Author Contributions

AY, NN, MG, IG, and RB contributed to conception and design of the study, database, and drafting of the manuscript. CB, EN, NB, MB, and WZ contributed to the database, the enrollment, and the drafting of the manuscript. All authors contributed to the manuscript revision, read, and approved the submitted version.

## Funding

This study was supported by a Research Grant from the Seymour Fefer Endowment.

## Conflict of Interest

The authors declare that the research was conducted in the absence of any commercial or financial relationships that could be construed as a potential conflict of interest.

## Publisher's Note

All claims expressed in this article are solely those of the authors and do not necessarily represent those of their affiliated organizations, or those of the publisher, the editors and the reviewers. Any product that may be evaluated in this article, or claim that may be made by its manufacturer, is not guaranteed or endorsed by the publisher.
