# Supplementary material for: QTc Dynamics Following Cardioversion for Persistent Atrial Fibrillation
Source: Front Cardiovasc Med. 2022 Jun 3;9:881446. doi: 10.3389/fcvm.2022.881446 (PMC9205203; doi:10.3389/fcvm.2022.881446)
Supplement: Supplementary file 1 [file Presentation_1.PPTX]

## Slide 1
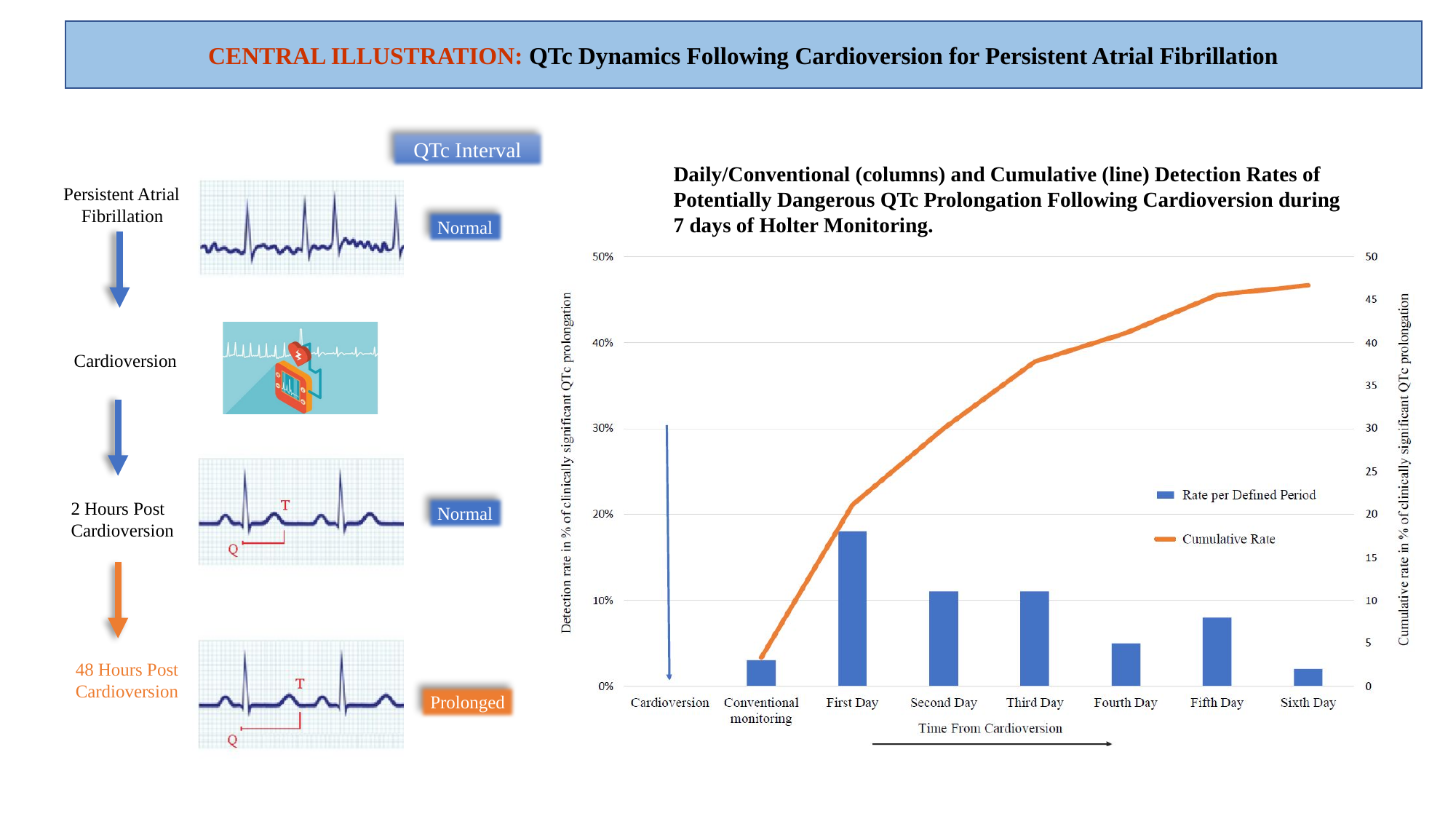

CENTRAL ILLUSTRATION: QTc Dynamics Following Cardioversion for Persistent Atrial Fibrillation
QTc Interval
Daily/Conventional (columns) and Cumulative (line) Detection Rates of Potentially Dangerous QTc Prolongation Following Cardioversion during 7 days of Holter Monitoring.
Persistent Atrial
 Fibrillation
Normal
Cardioversion
2 Hours Post
Cardioversion
Normal
48 Hours Post
Cardioversion
Prolonged
