# Supplementary material for: QTc Dynamics Following Cardioversion for Persistent Atrial Fibrillation
Source: Front Cardiovasc Med. 2022 Jun 3;9:881446. doi: 10.3389/fcvm.2022.881446 (PMC9205203; doi:10.3389/fcvm.2022.881446)

Online Appendix

QTc Dynamics Following Cardioversion for Persistent Atrial Fibrillation

Arwa Younis[1], MD, Nofrat Nehoray[2], MD, Michael Glikson[3], MD, Christopher Bodurian[4], BA, Eyal Nof[2], MD, Nicola Luigi Bragazzi[5], MD, PhD, MPH, Michael Berger[2], MD, Wojciech Zareba[4], MD, Ilan Goldenberg[4], MD, Roy Beinart[2], MD

1. Cardiac Electrophysiology & Pacing Section, Department of Cardiovascular Medicine, Cleveland Clinic, Cleveland, Ohio, United States.
2. Chaim Sheba Medical Center Affiliated to Sackler Medical School, Tel-Aviv University, Tel Hashomer, Israel.
3. Heart Center, Shaare Zedek Medical Center, Jerusalem, Israel.
4. Clinical Cardiovascular Research Center, University of Rochester, Rochester, New York United States.
5. Laboratory for Industrial and Applied Mathematics, Center for Disease Modelling, York University, Toronto, Canada.

Short title: QTc prolongation following cardioversion of AF

Corresponding Author:

Arwa Younis, MD
Cardiac Electrophysiology Section, Department of Cardiovascular Medicine
Cleveland Clinic
265 Crittenden Blvd., Box 653, Cleveland, OH 44120
E-mail: or.younis@gmail.com
Twitter: @arwayounis2
ORCID - 0000-0002-2485-5025

eTable A – List of the variables that were included in the predictors model.

Variable	Definition	HR	p value
Low LVEF	Left ventricular ejection fraction ≤ 40%	1.26 (0.54-2.95)	0.6
Age 65	Age ≥ 65 y	1.13 (057-2.23)	0.74
Male	Male gender	1.26 (0.62-2.55)	0.53
Renal Disease	Based on patients medical records	1.49 (0.69-3.2)	0.31
Hypertension	Based on patients medical records	1.21 (0.58-2.49)	0.61
Creatinine	Creatinine ≥ 1.5 mg/dl	1.19 (0.36-3.92)	0.77
Amiodarone	Prior* Amiodarone use	0.98 (0.47-2.01)	0.95
Beta blocker	Prior* Beta blocker use	6.59 (1.57-27.6)	<0.001
One C	Prior* use of Flecainide or Propafenone	1.05 (0.48-2.27)	0.91
Prior loading	Prior loading of antiarrhythmic medications	1.41 (0.71-2.79)	0.32
Reloading	Reloading of antiarrhythmic medications	0.26 (0.06-1.11)	0.12
QTC 450	Mean QTc ≥ 450 ms during first hour post ECV	0.91 (0.45-1.18)	0.075
Sinus hour one	SR in ECG one hour post ECV	1.36 (0.32-5.73)	0.67
High HR AF	HR ≥ 80 bpm during last hour prior CV#	1.10 (0.74-2.3)	0.41
High HR 1st	HR ≥ 70 bpm during first hour post CV#	1.13 (0.63-3.71)	0.37
High HR 2nd	HR ≥ 60 bpm during second hour post CV#	1.27 (0.53-2.49)	0.23
Mg 2	Magnesium ≥ 2 mg/dl#	1.21 (0.61-2.41)	0.59
K 4.7	Potassium ≥ 4.7 mg/dl#	0.82 (0.31-2.12)	0.67

* Patient has been taking the drug for more than 3 days.
# Cut based on the mean value.

Supplementary eTable B – Heart rate during 7-day Holter monitoring among those who prolonged the QTc significantly versus those who did not.

	Prolongation of the QTc*		
	Yes (N=39)<br>Median (IQR)	No (N=51)<br>Median (IQR)	p value
HR during AF	82 (66-88)	73 (63-94)	0.48
HR first hour after ECV	71 (59-79)	64 (57-75)	0.74
HR two hours after ECV	62 (52-72)	58 (55-64)	0.63
HR during Holter	69 (60-76)	63 (59-75)	0.33

* New prolongation of QTc ≥500 ms (if baseline QTc was < 480 ms), or prolongation of QTc ≥10% if baseline QTc was >480 ms. HR stands for heart rate, AF for atrial fibrillation or flutter, and ECV for electrical cardioversion.

eFigure A –Time-points for study measurements

		Baseline	Conventional monitoring	Holter monitoring													
Time (hours)	0	1	2	3	4	5	6		7	8	9	10		11	12	13	14
Patient 17	Cardioversion	429	423	425	431	460	452		496	469	435	439		436	431	437	441
Median QTc	NA	429	423	441					454					437			
Time for final analysis	0	1	2	4					8					12			

eFigure B1-B3 Electrocardiograms and Holter strips of study patient X in sinus rhythm following cardioversion:

B1) Electrocardiogram obtained within the first hour post cardioversion.

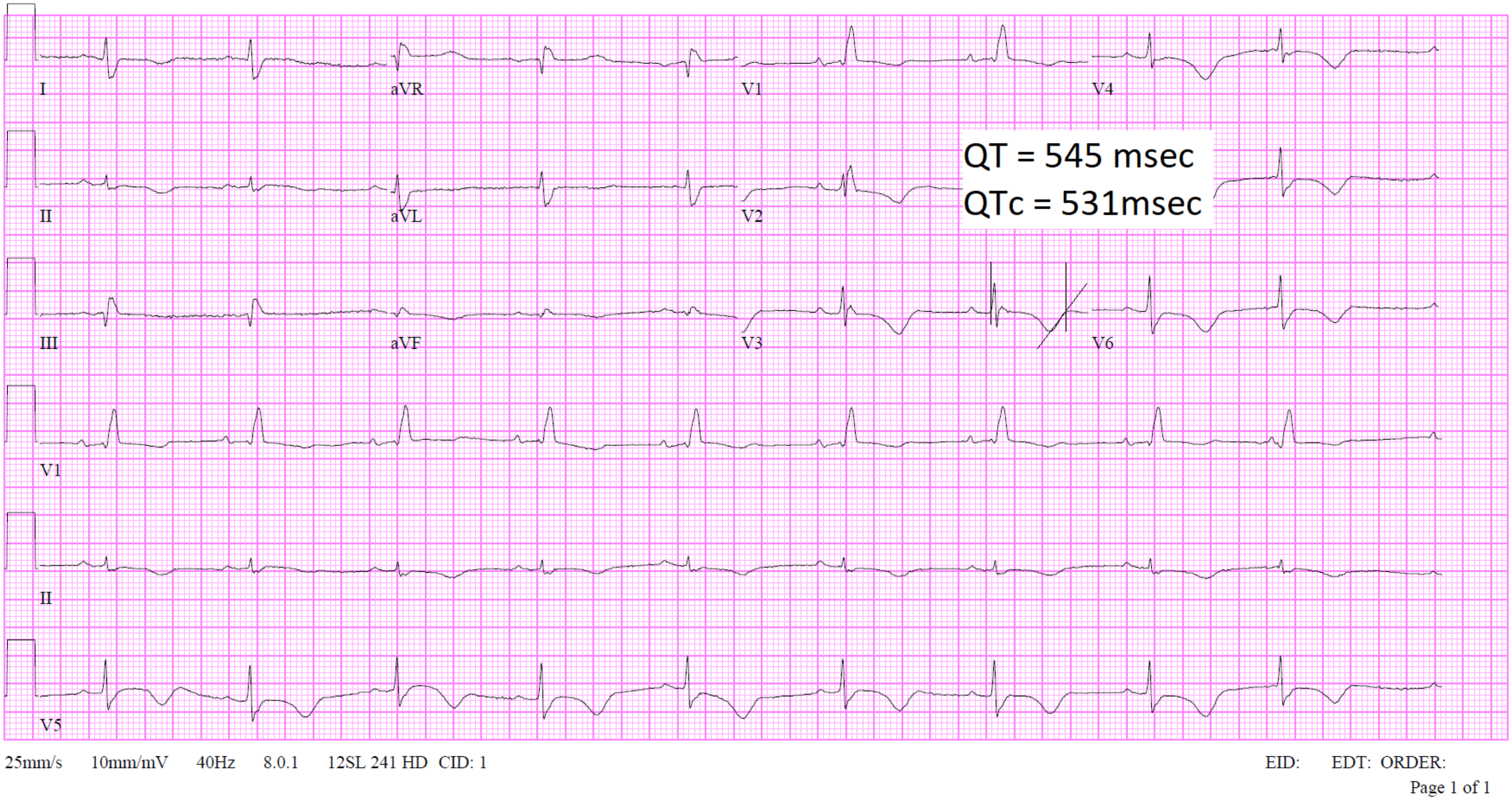

B2 – Holter strip three hours post cardioversion.

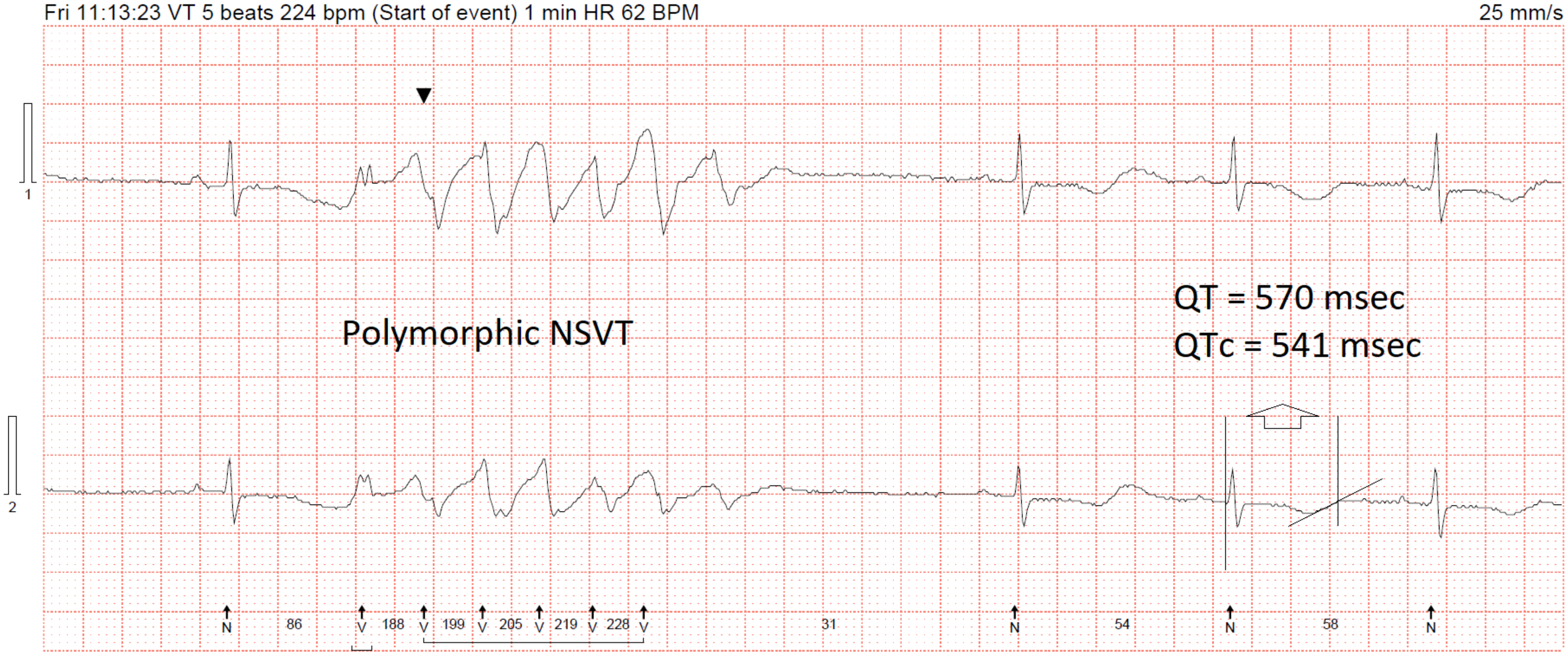

B3 – Holter strip 130 hour post CV.

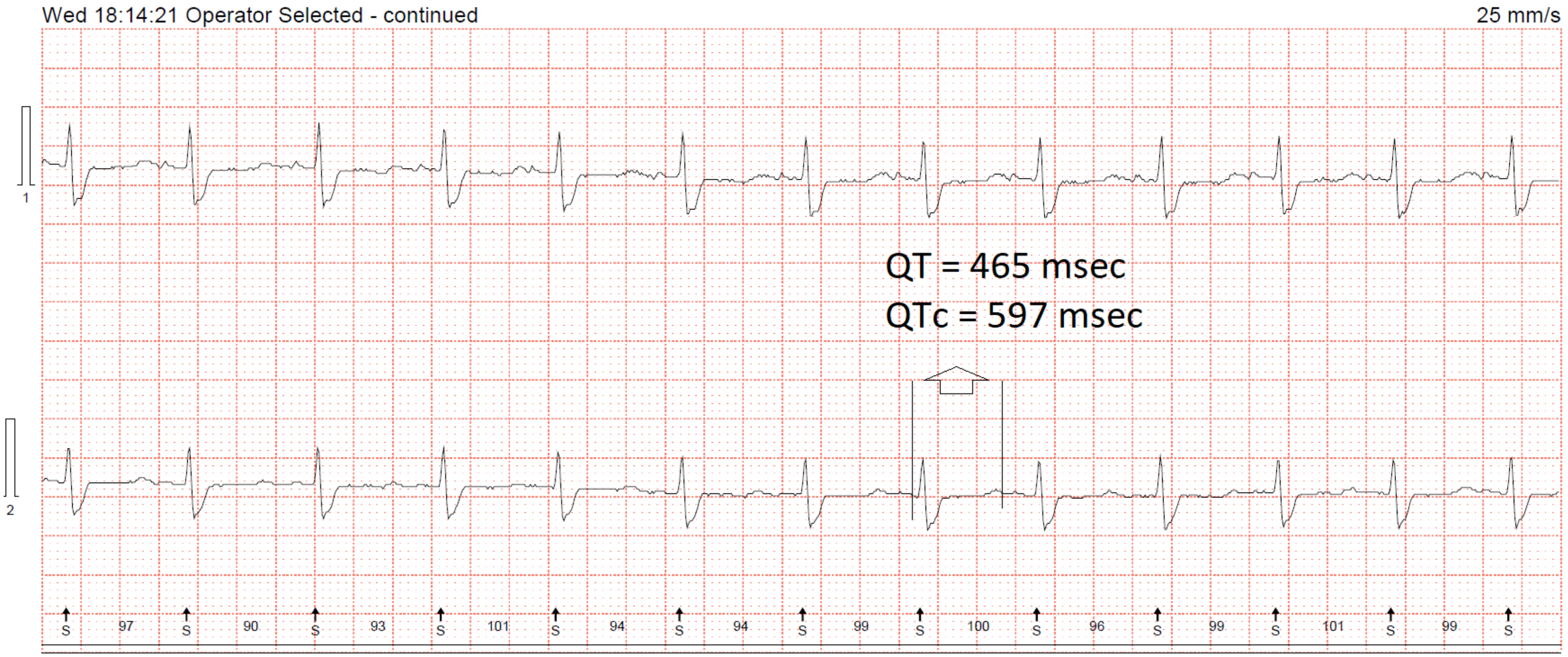

eFigure C1-C2 - Holter strips of study patient Y in sinus rhythm following cardioversion:

C1 – Holter strip within first hour post cardioversion.

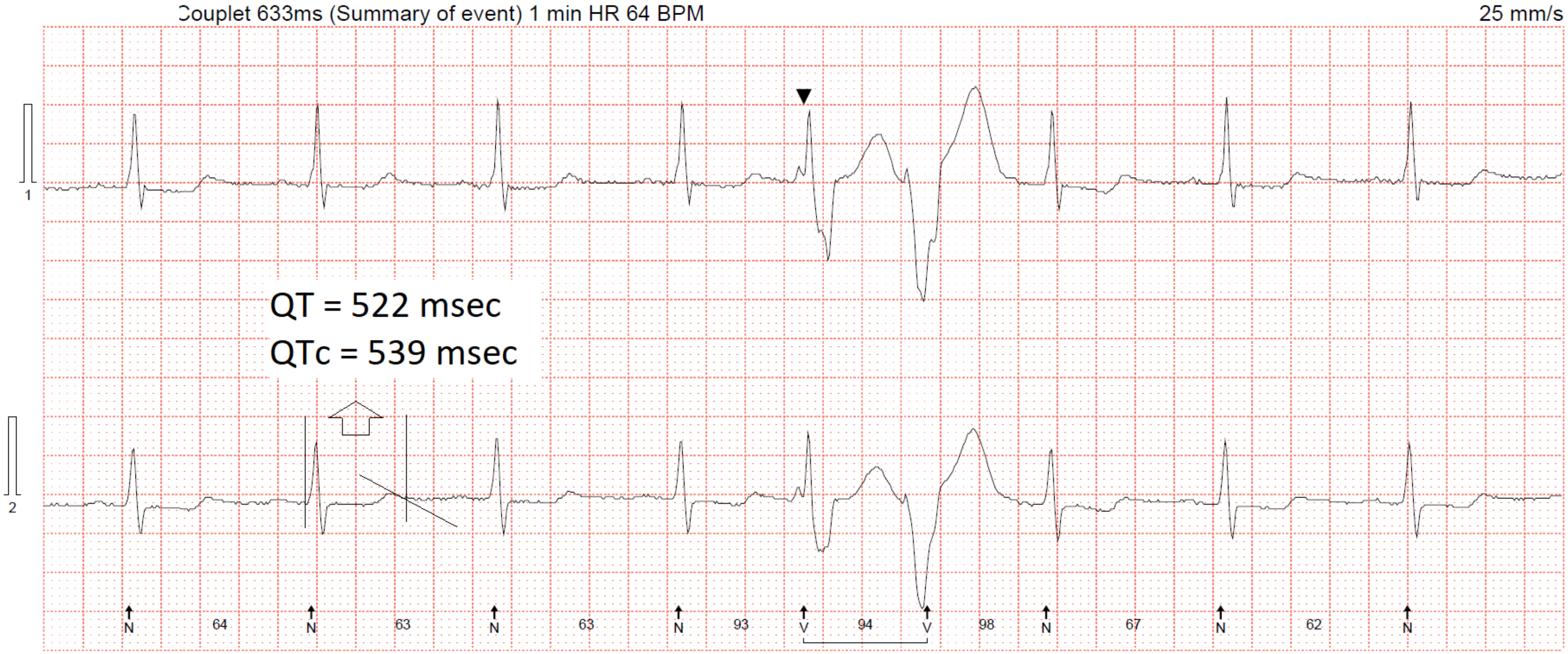

C2 – Holter strip 44 hour post cardioversion.

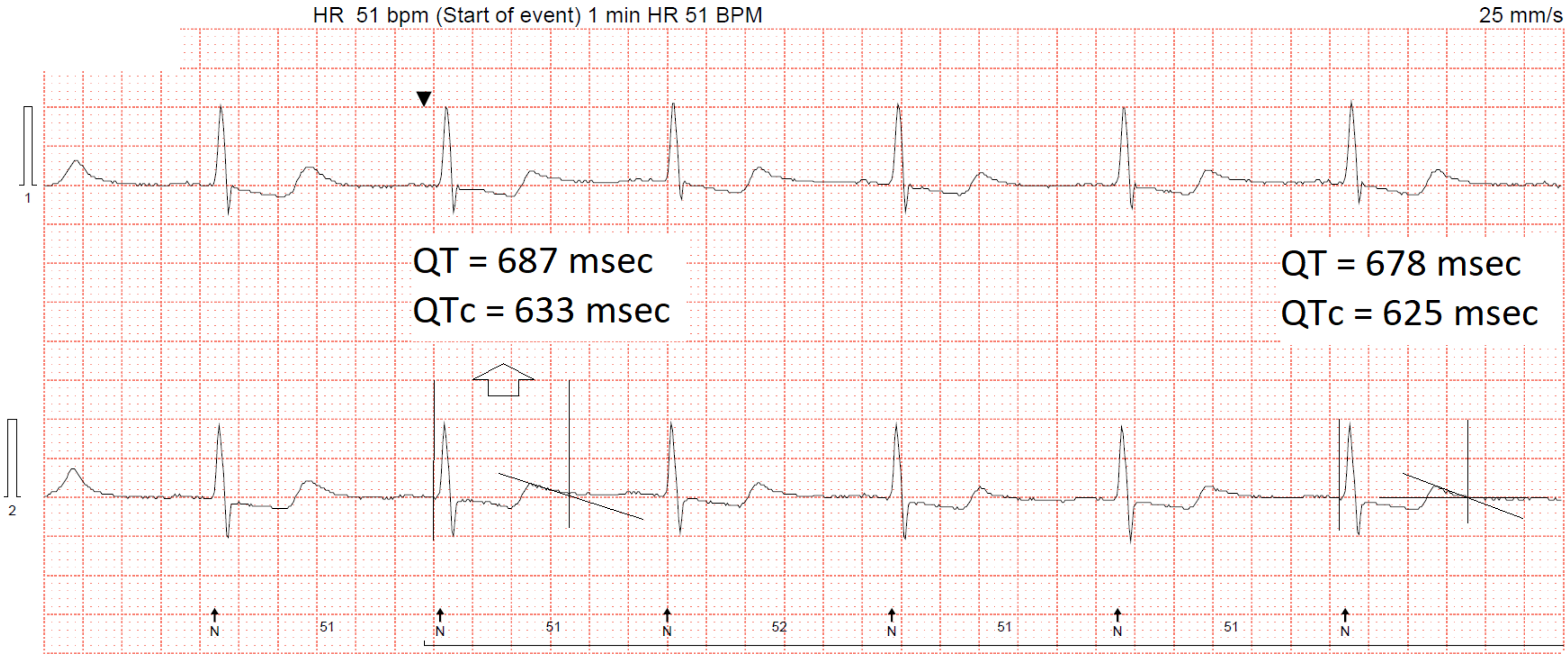

Supplement: Supplementary file 2 [file Data_Sheet_1.PDF]
